# The impact of comorbidities on prolonged mechanical ventilation in patients with chronic obstructive pulmonary disease

**DOI:** 10.1186/s12890-024-03068-9

**Published:** 2024-05-25

**Authors:** Kuang-Ming Liao, Hsueh-Yi Lu, Chung-Yu Chen, Lu-Ting Kuo, Bo-Ren Tang

**Affiliations:** 1 Department of Internal Medicine, Chi Mei Medical Center, Chiali, Taiwan; 2 Department of Nursing, Min-Hwei Junior College of Health Care Management, Tainan, Taiwan; 3 Department of Industrial Engineering and Management, National Yunlin University of Science and Technology, Yunlin, Taiwan; 4 School of Pharmacy, Kaohsiung Medical University, Kaohsiung, Taiwan; 5 Division of Neurosurgery, Department of Surgery, National Taiwan University Hospital, Taipei, Taiwan

**Keywords:** Chronic obstructive pulmonary disease, Comorbidity, Prolonged mechanical ventilation

## Abstract

**Background:**

In patients with chronic obstructive pulmonary disease (COPD) and acute respiratory failure, approximately 10% of them are considered to be at high risk for prolonged mechanical ventilation (PMV, > 21 days). PMV have been identified as independent predictors of unfavorable outcomes. Our previous study revealed that patients aged 70 years older and COPD severity were at a significantly higher risk for PMV. We aimed to analyze the impact of comorbidities and their associated risks in patients with COPD who require PMV.

**Methods:**

The data used in this study was collected from Kaohsiung Medical University Hospital Research Database. The COPD subjects were the patients first diagnosed COPD (index date) between January 1, 2012 and December 31, 2020. The exclusion criteria were the patients with age less than 40 years, PMV before the index date or incomplete records. COPD and non-COPD patients, matched controls were used by applying the propensity score matching method.

**Results:**

There are 3,744 eligible patients with COPD in the study group. The study group had a rate of 1.6% (60 cases) patients with PMV. The adjusted HR of PMV was 2.21 (95% CI 1.44–3.40; *P* < 0.001) in the COPD patients than in non-COPD patients. Increased risks of PMV were found significantly for patients with diabetes mellitus (aHR 4.66; *P* < 0.001), hypertension (aHR 3.20; *P* = 0.004), dyslipidemia (aHR 3.02; *P* = 0.015), congestive heart failure (aHR 6.44; *P* < 0.001), coronary artery disease (aHR 3.11; *P* = 0.014), stroke (aHR 6.37; *P* < 0.001), chronic kidney disease (aHR 5.81 *P* < 0.001) and Dementia (aHR 5.78; *P* < 0.001).

**Conclusions:**

Age, gender, and comorbidities were identified as significantly higher risk factors for PMV occurrence in the COPD patients compared to the non-COPD patients. Beyond age, comorbidities also play a crucial role in PMV in COPD.

## Background

Chronic obstructive pulmonary disease (COPD) is a systemic and chronic inflammatory disease characterized by airflow obstruction, lung parenchyma damage, and expiratory airflow limitation that is not fully reversible [1]. As COPD progresses, lung function gradually declines, and patients with COPD may experience respiratory failure. Furthermore, COPD patients with frequent exacerbations may be at risk of developing respiratory failure. In a previous study that enrolled patients requiring prolonged mechanical ventilation (more than 14 days) in the respiratory intensive care unit, it was found that the majority of these patients (59.3%) had COPD [2].

Another previous study, conducted over a one-year period and enrolling 327 patients requiring mechanical ventilation for more than 24 h, revealed that acute lung injury and COPD were the most prevalent conditions. The study found that the number of organ systems affected was associated with mortality. In patients with COPD and pneumonitis or retained secretions, mortality was lower (30%), but a significant percentage (43%) of these patients became ventilator-dependent. Ventilator dependence did not correlate with mortality during the course of respiratory failure [3].

A retrospective study conducted in a single hospital enrolled 87 COPD patients undergoing mechanical ventilation for acute respiratory failure due to non-surgical or traumatic events between January 1983 and December 1993. This study found that prolonged mechanical ventilation and COPD severity did not significantly affect the prognosis [4].

In studies focusing on patients with COPD and acute respiratory failure, it was observed that approximately 10% of them were at high risk for mechanical ventilation exceeding 21 days, and the weaning failure rate was high, ranging from 55 to 78%. Both weaning failure and prolonged mechanical ventilation (> 21 days) were identified as independent predictors of unfavorable outcomes in these patients [5–8].

The risk of prolonged mechanical ventilation in patients with COPD included advanced age, disease severity on admission, and the development of ventilator-associated pneumonia during the intensive care unit stay. A previous study found that patients aged 70 years and older were at a significantly increased risk for prolonged mechanical ventilation (> 21 days) compared to those aged 40–49 years. COPD severity was also associated with an increased risk of prolonged mechanical ventilation [9].

There is limited information available on the comparison of comorbidities in patients with COPD and prolonged mechanical ventilation. We hypothesized that different comorbidities and the number of comorbidities could influence the risk of prolonged mechanical ventilation in COPD. Our aim was to analyze the comorbidities and their impact on the risk of prolonged mechanical ventilation in patients with COPD.

## Methods

### Ethics statement

This study underwent review by the Institutional Review Board (IRB) of Kaohsiung Medical University Hospital, Taiwan (IRB no. KMUHIRB-E(I)-20220124). Informed consent was waived by the approving IRB, and all personally identifiable information was meticulously de-identified from the dataset to ensure strict anonymity.

### Data sources

The data utilized in this study was obtained from the Kaohsiung Medical University Hospital Research Database (KMUHRD), encompassing inpatient and outpatient registration, as well as claims data from one tertiary teaching hospital and two secondary medical institutions within the same medical system in Taiwan. This dataset includes patients' demographic characteristics, disease diagnoses, surgery operation codes (based on the International Classification of Diseases, Ninth Revision, Clinical Modification [ICD-9-CM]), outpatient and inpatient admission details, prescription records, and medical expenditures. For this study, we retrospectively employed a longitudinal dataset spanning from 2013 to 2020.

### Patients

**Fig. 1:**
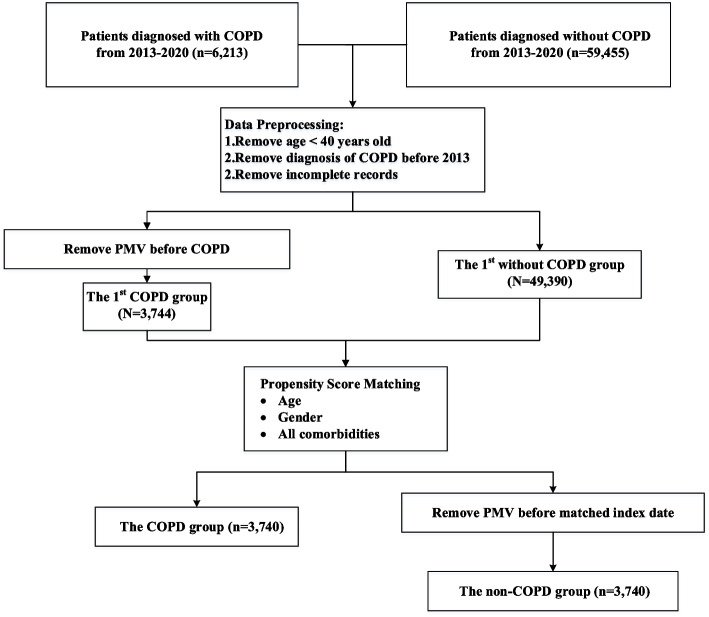
Flowchart of subject enrollment

The study included COPD subjects who were initially diagnosed with ICD-9-CM codes 490–492 and 496 between January 1, 2012, and December 31, 2020. Each eligible COPD patient had to have at least three outpatient visits or one inpatient admission with a primary diagnosis of COPD in the KMUHRD records. The index date, the earliest date of the third visit or inpatient admission, was assigned for assessing the risk of prolonged mechanical ventilation. To ensure the first-time diagnosis of COPD, selected patients were required to have no COPD codes in their records for one year before the index date. Initially, 6,213 COPD patients were eligible before applying exclusion criteria (see Fig. 1). Exclusion criteria encompassed patients under 40 years of age, those not receiving a first-time COPD diagnosis, individuals undergoing prolonged mechanical ventilation before the index date, malignancy and those with incomplete records. The index date for the COPD group was defined as the date of first diagnosis. The non-COPD group consisted of individuals identified from the KMUHRD who did not have COPD and were matched to the COPD patients based on the same index date.

#### Propensity score matching

Control group subjects were randomly selected from patients without a COPD diagnosis in the KMUHRD database. To ensure homogeneity in baseline characteristics between COPD and non-COPD patients, we employed propensity score matching. Propensity score matching is a statistical method designed to mitigate the confounding effects of non-random sampling experiments. The propensity score was estimated through a probability function based on a multivariable logistic regression model to reduce selection bias regarding covariates between COPD and non-COPD groups [10]. These covariates included age, sex, diabetes mellitus, hypertension, dyslipidemia, congestive heart failure, coronary artery disease, stroke, chronic kidney disease, cirrhosis, and dementia. Control subjects were matched and selected with a propensity score within ± 0.05 standard deviation at a 1:1 ratio.

### Outcomes and comorbidities

To assess the risk of prolonged mechanical ventilation, patients were followed from the index date until the occurrence of prolonged mechanical ventilation, death, loss to follow-up, or the end of 2020, whichever came first. The death records were obtained from the electronic medical records of the medical center, and this database provides the exact date of death.

Prolonged mechanical ventilation was defined as either invasive mechanical ventilation for 21 or more days, invasive mechanical ventilation followed by non-invasive ventilation with a total duration of 21 or more days, invasive mechanical ventilation followed by negative pressure ventilation with a total duration of 21 or more days, or requiring non-invasive ventilation for 21 or more days.

Comorbidities, including diabetes mellitus (ICD-9-CM code 250), hypertension (ICD-9-CM code 401–405), dyslipidemia (ICD-9-CM code 272.4), congestive heart failure (ICD-9-CM code 402.01, 402.11, 402.91, 425, 428, 429.3), coronary artery disease (ICD-9-CM code 410–414), stroke (ICD-9-CM code 430–438), chronic kidney disease (ICD-9-CM code 585), liver cirrhosis (including cirrhosis, ICD-9-CM code 571.5, and 571.6), and dementia (ICD-9-CM code 290), were identified from records within one year before the index date in the outpatient and inpatient records.

### Statistical analysis

Demographic and comorbidity variables for COPD and non-COPD patients were expressed as frequencies (percentages) or means (± standard deviation, SD). Group comparisons were conducted using chi-square tests and Student's t-test. Demographic characteristics included gender and age, stratified into age groups: 40–49, 50–59, 60–69, 70–79, and over 80 years. Cumulative incidence curves for prolonged mechanical ventilation were generated using the Kaplan–Meier method, and differences in the curves between COPD and non-COPD groups were evaluated using a log-rank test. The total follow-up period (TFP) was calculated as the sum of follow-up years (from the index date to either the occurrence of prolonged mechanical ventilation or the end of follow-up) for all patients in each group. The incident rate of prolonged mechanical ventilation was estimated by dividing the total number of prolonged mechanical ventilation events by the TFP and multiplying by 1,000 (per 1,000 person-years). Cox proportional hazards models were utilized to estimate the main effect of comorbidities for COPD patients regarding the occurrence of prolonged mechanical ventilation. Hazard ratios (HRs) and their corresponding 95% confidence intervals (CIs) were estimated using Cox regression. All variables were included in the Cox multivariable regression model. All statistical tests were two-sided, and a *P*-value of 0.05 was considered statistically significant.

## Results

### Patient characteristics

Figure 1 shows the flowchart of subject enrollment during the date preprocessing and propensity score matching. Following the data removal process, 3,744 eligible patients with COPD were selected for the study group and 49,390 ones for without COPD group (indicated 1st in Fig. 1). Table 1 shows the demographic characteristics and comorbidities of all patients among COPD and the control (non-COPD) groups in the original dataset. Propensity scores were calculated based on covariate variables, including age, gender, and comorbidities associated with prolonged mechanical ventilation. After propensity score matching, the non-COPD comprised an equal number of patients, totaling 3,740. In the study group, 1.6% of patients (60 cases) experienced prolonged mechanical ventilation, while the control group exhibited a rate of 0.88% for prolonged mechanical ventilation (see Table 2). Table 2 provides a comparison of the basic characteristics between the COPD and non-COPD groups after propensity score matching. Both age, gender, and comorbidity variables were evenly distributed within both groups, thereby enhancing comparability.

**Table 1:** Demographic characteristics and comorbidities of patients before PSM

Variables	COPD (*n* = 3,740)	Non-COPD (*n* = 49,390)	Standardized mean difference
Age (%)			
Mean (± SD)	65.96(11.83)	60.26(12.11)	0.477
Gender (%)			
Male	2,149(57.4)	23,517(47.61)	0.197
Female	1,595(42.6)	25,873(52.39)	
Comorbidities (%)			
Diabetes mellitus	1,105(29.51)	11,396(23.07)	0.147
Hypertension	2,000(53.42)	19,562(39.61)	0.280
Dyslipidemia	792(21.15)	6,383(12.92)	0.220
Congestive heart failure	503(13.43)	2,791(5.65)	0.267
Coronary artery disease	665(17.76)	4,245(8.59)	0.274
Stroke	556(14.85)	4,128(8.36)	0.204
Chronic kidney disease	556(14.85)	4,167(8.44)	0.201
Liver cirrhosis	183(4.89)	2,659(5.38)	0.022
Dementia	237(6.33)	1,292(2.62)	0.180

*PSM* propensity score matching

**Table 2:** Demographic characteristics and comorbidities of patients after PSM

Variables	COPD (*n* = 3,740)	Non-COPD (*n* = 3,740)	Standardized mean difference
Age (%)			
40–49	368(9.84)	360(9.63)	
50–59	716(19.14)	724(19.36)	
60–69	1,215(32.49)	1,200(32.09)	
70–79	931(24.89)	970(25.94)	
≥ 80	510(13.64)	486(12.99)	
Mean (± SD)	65.94(11.82)	65.94(11.76)	< 0.01
Gender (%)			
Male	2,145(57.35)	2,171(58.05)	< 0.01
Female	1,595(42.65)	1,569(41.95)	
Comorbidities (%)			
Diabetes mellitus	1,104(29.52)	1,101(29.44)	< 0.01
Hypertension	1,996(53.37)	2,041(54.57)	< 0.01
Dyslipidemia	790(21.12)	815(21.79)	< 0.01
Congestive heart failure	499(13.34)	484(12.94)	< 0.01
Coronary artery disease	661(17.67)	648(17.33)	< 0.01
Stroke	552(14.76)	538(14.39)	< 0.01
Chronic kidney disease	552(14.76)	557(14.89)	< 0.01
Liver cirrhosis	183(4.89)	167(4.47)	< 0.01
Dementia	234(6.26)	194(5.19)	< 0.01
**Outcomes (%)**			
PMV	60(1.60)	33(0.88)	

*PSM* propensity score matching, *PMV* prolonged mechanical ventilation

### Incidence of prolonged mechanical ventilation

In Table 3, the COPD group displayed a higher overall incidence rate of prolonged mechanical ventilation compared to the non-COPD group (3.99 vs. 1.62 per 1,000 person-years). The adjusted hazard ratio (aHR) of 2.21 (95% CI 1.44–3.40; *P* < 0.001) indicated that the risk of experiencing prolonged mechanical ventilation was 2.21 times higher for patients in the COPD group than for those in the non-COPD group. This relationship was further explored by analyzing the associations between age, gender, and comorbidities. When only looking at the COPD group in Table 3, the incidence rates (IR) in the stratified age groups (40–49, 50–59, 60–69, 70–79, and over 80 years) increased (IR = 1.97, 1.65, 4.28, 4.53, and 7.26 respectively). With increasing age, the incidence of PMV also increases. These incidence rates were also significantly higher than those in the non-COPD group for all variables. After Cox regression, it was found that age, gender, and comorbidities posed significantly higher risks of prolonged mechanical ventilation occurring in the COPD group compared to the non-COPD group (refer to Table 3). For instance, for patients with diabetes mellitus, the adjusted HR for prolonged mechanical ventilation in the COPD group was 3.10 (95% CI 1.67–5.76; *P* < 0.001) times higher than that in the non-COPD group.

**Table 3:** Incidence of prolonged mechanical ventilation in patients with and without COPD

Characteristics	COPD(*n* = 3,740)			Non-COPD(*n* = 3,740)			aHR(95%CI)	*P*-value
	Event	TFP(year)	IR	Event	TFP(year)	IR		
PMV	60	15054	3.99	33	20395	1.62	2.21(1.44–3.40)	< 0.001
Age								
40–49	3	1524.38	1.97	1	1827.49	0.55	3.18(0.33–30.60)	0.317
50–59	5	3036	1.65	4	3964.01	1.01	1.34(0.36–5.01)	0.660
60–69	20	4673.49	4.28	9	6404.02	1.41	2.57(1.16–5.67)	0.020
70–79	17	3756.11	4.53	14	5471.53	2.56	1.74(0.84–3.58)	0.135
80 and above	15	2064.82	7.26	5	2728.5	1.83	3.46(1.25–9.57)	0.017
Gender								
Male	38	8548.85	4.45	22	11831.88	1.86	2.16(1.27–3.68)	0.004
Female	22	6505.94	3.38	11	8563.66	1.28	2.34(1.13–4.84)	0.023
Diabetes mellitus								
Yes	34	4410.68	7.71	15	6476.92	2.32	3.10(1.67–5.76)	< 0.001
No	26	10644.11	2.44	18	13918.63	1.29	1.62(0.89–2.96)	0.116
Hypertension								
Yes	43	8188.7	5.25	24	11806.73	2.03	2.31(1.4–3.84)	0.001
No	17	6866.09	2.48	9	8588.82	1.05	2.11(0.93–4.78)	0.074
Dyslipidemia								
Yes	16	3239.18	4.94	9	4999.86	1.8	2.63(1.14–6.04)	0.023
No	44	11815.62	3.72	24	15395.68	1.56	2.11(1.28–3.48)	0.004
Congestive heart failure								
Yes	22	2161.86	10.18	10	2959.65	3.38	2.73(1.28–5.81)	0.009
No	38	12892.93	2.95	23	17435.9	1.32	1.98(1.17–3.34)	0.011
Coronary artery disease								
Yes	14	2846.72	4.92	6	4074.16	1.47	2.89(1.09–7.62)	0.032
No	46	12208.08	3.77	27	16321.38	1.65	2.06(1.28–3.33)	0.003
Stroke								
Yes	24	2365.68	10.15	13	3363.63	3.86	2.41(1.21–4.79)	0.012
No	36	12689.12	2.84	20	17031.91	1.17	2.11(1.22–3.65)	0.008
Chronic kidney disease								
Yes	22	2329.19	9.45	11	3332.02	3.3	2.84(1.36–5.95)	0.006
No	38	12725.6	2.99	22	17063.52	1.29	1.96(1.16–3.32)	0.013
Liver cirrhosis								
Yes	3	765.46	3.92	1	968.75	1.03	3.12(0.32–30.05)	0.324
No	57	14289.33	3.99	32	19426.8	1.65	2.18(1.41–3.38)	< 0.001
Dementia								
Yes	9	994.49	9.05	6	1199.81	5	1.84(0.64–5.30)	0.257
No	51	14060.3	3.63	27	19195.73	1.41	2.25(1.41–3.60)	< 0.001

*Abbreviations*: *COPD* chronic obstructive pulmonary disease, *TFP* total follow-up period, *IR* incident rate per 1000 person-year, *aHR* adjusted hazard ratio, *CI* confidence interval

### Impact of comorbidities on the occurrence of prolonged mechanical ventilation

The association between comorbidities and the occurrence of prolonged mechanical ventilation in COPD patients was further investigated (see Table 4). The baseline group consisted of COPD patients without any comorbidities, and their risk of prolonged mechanical ventilation was compared to those with various comorbidities. It was found that patients with diabetes mellitus (aHR 4.66; *P* < 0.001), hypertension (aHR 3.20; *P* = 0.004), dyslipidemia (aHR 3.02; *P* = 0.015), congestive heart failure (aHR 6.44; *P* < 0.001), coronary artery disease (aHR 3.11; *P* = 0.014), stroke (aHR 6.37; *P* < 0.001), chronic kidney disease (aHR 5.81; *P* < 0.001), and dementia (aHR 5.78; *P* < 0.001) had significantly increased risks of prolonged mechanical ventilation. COPD patients with at least one comorbidity were also found to have a significantly higher risk of prolonged mechanical ventilation (aHR 2.96; *P* = 0.007) compared to those with no comorbidity. Similar results were observed for patients with at least two comorbidities (aHR 3.75; *P* = 0.001) and at least three comorbidities (aHR 5.21; *P* < 0.001).

**Table 4:** Impact of comorbidities on prolonged mechanical ventilation among COPD patients

Comorbidity	Number (*n* = 3740)	Even	aHR (95% CI)	*P*-value
No comorbidity	1061(28.4)	7(0.7)	-	-
Diabetes mellitus	1104(29.5)	34(3.1)	4.66(2.07–10.51)	< 0.001
Hypertension	1996(53.4	43(2.2)	3.20(1.44–7.11)	0.004
Dyslipidemia	790(21.1)	16(2.0)	3.02(1.24–7.33)	0.015
Congestive heart failure	499(13.3)	22(4.4)	6.44(2.75–15.01)	< 0.001
Coronary artery disease	661(17.7)	14(2.1)	3.11(1.25–7.70)	0.014
Stroke	552(14.8)	24(4.3)	6.37(2.74–14.78)	< 0.001
Chronic kidney disease	552(14.8)	22(4.0)	5.81(2.48–13.61)	< 0.001
Liver cirrhosis	183(4.9)	3(1.6)	2.46(0.64–9.50)	0.193
Dementia	234(6.3)	9(3.8)	5.78(2.08–14.98)	0.001
≥ 1 comorbidities	2679(71.6)	53(2)	2.96(1.34–6.50)	0.007
≥ 2 comorbidities	1816(48.6)	46(2.5)	3.75(1.69–8.29)	0.001
≥ 3 comorbidities	1104(29.5)	39(3.5)	5.21(2.33–11.64)	< 0.001

### Cumulative incidence rates of prolonged mechanical ventilation

**Fig. 2:**
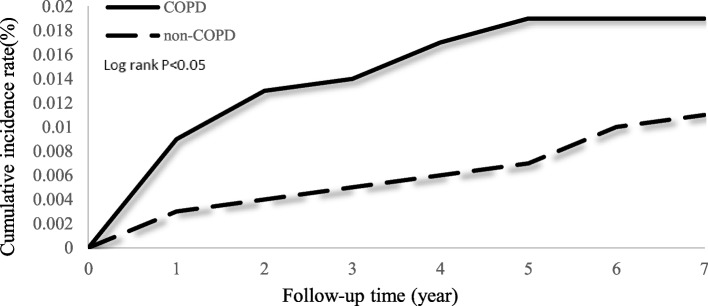
Cumulative incidence of prolonged mechanical ventilation in patients with and without COPD

**Fig. 3:**
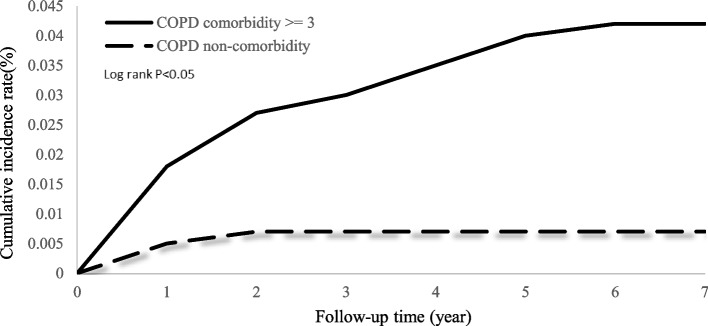
Cumulative incidence of prolonged mechanical ventilation in patients with ≥ 3 comorbidity

Cumulative incidence curves depicting the occurrences of prolonged mechanical ventilation over time revealed significant differences (*P* < 0.05, according to the log-rank test) between the COPD and non-COPD groups (see Fig. 2). In Fig. 3, COPD patients with at least three comorbidities were significantly associated with a higher risk of prolonged mechanical ventilation occurring when compared to COPD patients with no comorbidity (*P* < 0.05, by log-rank test).

## Discussion

Anticipating the probability of prolonged mechanical ventilation in COPD patients, considering their comorbidities, is a complex task. It often relies on subjective factors such as the patient's clinical state, the severity of the disease, the cause of admission, the presence of respiratory failure, and lung function. So far, there have been no established tools to accurately predict the likelihood of prolonged mechanical ventilation in COPD patients following intubation, which could help guide physicians and families in making informed decisions at a later stage. In our research, we aim to utilize data from our COPD patient population, including their comorbidities, to forecast the potential risk of prolonged mechanical ventilation. Increased risks of prolonged mechanical ventilation were found significantly for COPD patients with comorbidities included diabetes mellitus, hypertension, dyslipidemia, congestive heart failure, coronary artery disease, stroke, chronic kidney disease and dementia. The higher the number of comorbidities in patients with COPD, the greater the risk of prolonged mechanical ventilation.

### Definition of prolonged mechanical ventilation

The definition of prolonged mechanical ventilation varies across different studies, ranging from periods exceeding 24 h to longer than 29 days [3, 11]. Additionally, there is sometimes confusion between prolonged weaning and prolonged mechanical ventilation, with the terms used interchangeably [12]. The WIND study proposed a definition for prolonged weaning as a situation where weaning had not been successfully completed seven days after the initial separation attempt [13].

The Society of Thoracic Surgeons, on the other hand, defines mechanical ventilation for a duration exceeding 24 h as prolonged mechanical ventilation [14]. In our study, we adopted a criterion of more than 21 days of mechanical ventilation, where each day required at least 6 h of ventilation [15].

Furthermore, within the framework of Taiwan's National Health Insurance program, patients requiring prolonged mechanical ventilation have the option to apply for catastrophic illness certification. This certification exempts them from certain National Health Insurance payments and copayments for each healthcare encounter. All applications for catastrophic illness certification undergo examination by experts, ensuring a very high degree of accuracy in the diagnosis of catastrophic illness [16].

### Comorbidities was associated with a poor outcome and prolonged mechanical ventilation: heart failure, stroke, renal function

Patients with COPD often experience various comorbidities, including cardiovascular and cerebrovascular diseases. In a multicenter registry in the USA comprising 1,664 ambulatory COPD patients, the prevalence of heart failure (HF), confirmed by medical record review, was reported at 15.7% [17]. Multivariate analysis revealed that heart failure was associated with an increased risk of mortality, with an adjusted hazard ratio of 1.33 (95% confidence interval: 1.06–1.68) [17], indicating a statistically significant link between heart failure and mortality among COPD patients. Additionally, a study utilizing the BODE cohort enrolled patients with COPD between January 1997 and December 2015 [18]. Authors included 2,145 participants with COPD, found that 14.6% had heart failure. The adjusted hazard ratio for mortality associated with heart failure in this group was 1.34 (95% confidence interval: 1.09–1.67) [19], further substantiating the significant impact of heart failure on mortality in COPD patients.

A study, limited by its single-center, retrospective design, involved 670 patients diagnosed with severe COPD who required mechanical ventilation for acute respiratory failure; it found that only 4% of these patients had heart failure [20]. Cardiac dysfunction is often a primary reason for unsuccessful weaning from prolonged mechanical ventilation. Specifically, patients with heart failure face a heightened risk of weaning-induced cardiac dysfunction. When undergoing prolonged mechanical ventilation, those with cardiovascular diseases demonstrate a lower rate of successful weaning compared to patients with other causes. During the weaning process, as ventilator support decreases, patients with COPD experience an increase in the work of breathing, leading to respiratory distress, elevated sympathetic tone, and increased myocardial aerobic metabolism and workload [21].

Patients experiencing stroke may face challenges such as impaired consciousness, hypoventilation, or aspiration pneumonia, leading to respiratory failure and the risk of prolonged mechanical ventilation [22, 23]. Research indicates that between 25 and 33% of individuals with stroke require intubation and mechanical ventilation due to respiratory failure [24, 25]. Approximately 10% of stroke survivors end up needing prolonged mechanical ventilation [24, 25]. Hospital mortality rates are notably high for those requiring ventilation following an acute stroke, with 65–70% of these patients experiencing severe coma (Glasgow Coma Scale score < 8) [25]. Although some stroke patients may see an improvement in consciousness during their hospital stay, lower Glasgow Coma Scale scores or ongoing impairment in consciousness at the weaning center pose significant risks for weaning failure [26]. For patients with neurological disorders, impaired brain function increases the risk of needing prolonged mechanical ventilation. Patients who continue to exhibit a lack of consciousness find it difficult to be weaned off mechanical ventilation. Additionally, this compromised state of consciousness hampers their ability to protect their airways, heightening the likelihood of aspiration after they are extubated. Consequently, persistent impairment in consciousness among stroke patients often leads to weaning failure and can extend the period of mechanical ventilation.

Charson et al. [27] employed predictive variables that were measured on the 21st day of ventilation to predict mortality. These variables included the need for vasopressors, hemodialysis, a platelet count of 150 × 109/L or lower, and an age of 50 years or older. They utilized these variables to create predictive models for both one-year and three-month mortality and mortality rates closely aligned with the predicted mortality rates for both the three-month and twelve-month periods. These four predictive variables can be effectively employed as a prognostic scoring system to differentiate between low-risk and high-risk patients. Low-risk patients, who exhibit none of these risk factors, had a relatively low 15% mortality rate. In contrast, high-risk patients, characterized by the presence of three or all four risk factors, faced a substantially higher 97% mortality rate. Authors aimed to delineate clinical factors at the time of intubation that could serve as indicators for individuals with prolonged mechanical ventilation in medical intensive care unit [28]. In their study, prolonged ventilation was defined as requiring ventilatory support for more than 14 days. The key variables independently linked with prolonged mechanical ventilation were identified as the need for intubation after admission to the intensive care unit, a heart rate exceeding 110 beats per minute, a blood urea nitrogen level exceeding 25 mg/dL, a serum pH level below 7.25, a creatinine level > 2.0 mg/dL, and a bicarbonate level lower than 20 mEq/L. The specificity of predicting prolonged ventilation was found to be 100% when four or more of these variables were present. Renal function plays a crucial role, not only in predicting mortality among patients undergoing prolonged ventilation but also in assessing the risk of prolonged ventilation in the medical intensive care unit. Our study further revealed that patients with COPD who also had chronic renal disease were at significantly higher risk, with an adjusted hazard ratio of 5.81, for experiencing prolonged ventilation (> 21 days) when compared to COPD patients without chronic kidney disease.

### Laboratory data and dependence on a ventilator

In addition to age, previous studies have also incorporated ventilator parameters, such as the oxygenation index (calculated as the mean airway pressure multiplied by FiO2 divided by PaO2), as well as blood pressure and the use of vasopressors, to predict the likelihood of mortality and/or dependence on a ventilator for a period exceeding 14 days [29, 30]. A systematic review unveiled that risk factors relevant to prolonged ventilation encompassed various elements. These encompassed age [13, 31] and comorbidities like previous stroke, chronic kidney disease, heart failure, and COPD [30, 32–35]. Additionally, several laboratory parameters were identified, including platelet counts, hypernatremia, blood urea nitrogen, blood glucose levels, creatinine levels, and serum albumin [28, 30, 31, 33, 35, 36]. Parameters associated with ventilator settings and blood gas values, along with facets of gas exchange (e.g., PaO2/FiO2 < 200 mm Hg) [30, 31, 37] were also considered relevant factors.

Previous studies have aimed to identify predictors of prolonged mechanical ventilation. Emphasis has been placed on laboratory data and arterial blood gas analyses in these investigations. Nevertheless, the task of pinpointing a definitive set of critical predictors remains challenging, mainly due to variations in the clinical characteristics of patients and diverse clinical settings. Furthermore, the absence of a consensus definition for prolonged ventilation has contributed to the complexity of these studies. In our study, comorbidities are being examined as potential factors that may influence the risk of ventilator dependence.

### Comorbidities and dependence on a ventilator in COPD

Comorbidities inpatients with COPD have received less attention compared to other parameters like laboratory data, ventilator settings, and arterial blood gas in previous report. It's important to consider comorbidities when assessing the likelihood of ventilator dependence in patients with COPD because these additional health conditions can significantly impact a patient's overall health and respiratory function. Comorbidities such as heart disease, diabetes, or other kidney diseases can complicate the clinical picture and make it more challenging to predict a patient's response to ventilator therapy and their potential for weaning from the ventilator.

We provide information that could help physicians better understand the complexities of managing ventilator-dependent patients with COPD and make more informed decisions about their care. It's also worth noting that the timing of laboratory data collection and analysis in COPD patients with respiratory failure is indeed crucial, as disease severity and patient status can change over time.

Our study specifically focuses on patients with COPD and investigates the association between patient comorbidities and prolonged ventilation (> 21 days). We found that COPD patients with heart failure and stroke face more than a six-fold risk of prolonged ventilation, while those with chronic kidney disease and dementia have more than a five-fold risk of prolonged ventilation compared to those without these comorbidities. Frequent and comprehensive monitoring of patients with COPD and their comorbidities, as well as considering other relevant parameters, can be essential for determining the most appropriate treatment strategies and for assessing the potential for ventilator weaning. Our study can contribute to the ongoing efforts to improve patient care in this context.

According to our previous study, patients with COPD aged 70 years and older were significantly more likely to become dependent on mechanical ventilation compared with individuals aged 40–49 years. After adjusting for confounding factors, we found that the severity of COPD was indicated by the costs per visit and the annual number of hospital visits, both of which were associated with an increased risk of prolonged mechanical ventilation [9].

Additionally, a retrospective study utilizing the longitudinal National Health Insurance Research Database in Taiwan assessed the long-term mortality and associated medical costs among patients requiring prolonged mechanical ventilation, comparing those with tracheostomies to those without. This study tracked patients for five years to assess overall survival rates. The average survival period was 4.98 years for patients with tracheostomies and 5.48 years for those undergoing translaryngeal intubation, although the difference was not statistically significant. COPD was identified as a significant factor contributing to mortality in patients with endotracheal intubation. The literature indicates that COPD and pneumonia are two common reasons for ventilator dependency. Patients with COPD were not only found to have difficulty being weaned off ventilators, but COPD was also recognized as an independent risk factor for the long-term mortality of ventilator-dependent patients [38]. Our study found that comorbidities in COPD patients impacted the duration of mechanical ventilation. The more comorbidities COPD patients had, the higher their risk of experiencing prolonged mechanical ventilation. Comorbidities in COPD were not only independent predictors of one-year mortality in COPD patients [39] but also had a significant influence on ventilator dependence.

### Limitation

Our study has certain limitations. Firstly, our electronic medical database lacked lung function data and challenging to assess the dyspnea score of COPD patients within our population. Consequently, we were unable to determine the stage and severity of COPD in our study participants. Secondly, the significance of comorbidity severity, such as diabetes and heart failure, in contributing to respiratory failure and prolonged mechanical ventilation, was not explored in terms of different severity levels among patients. We also acknowledge that we do not know whether the study outcome (prolonged mechanical ventilation) would remain the same if these comorbidities were well-controlled. It is crucial to advance our clinical practice by actively managing these comorbidities, rather than merely being aware of them. This consideration has been added to our study’s limitations.

Thirdly, our data were derived from a medical center and regional hospitals in southern Taiwan. Therefore, it is advisable to approach the generalizability of these results to other populations in different countries with caution. Furthermore, our database lacks certain physiological data, such as temperature, mean arterial pressure, pH, heart rate, and respiratory rate. Consequently, we were unable to evaluate the APACHE II Score before patients received mechanical ventilation.

Sarcopenia and cachexia are well-known for being associated with poor outcomes in COPD, but their coding has seen little use in clinical settings and has not undergone further validation in our database. Patients with malignancies also need to consider the impact of competing risks; therefore, we excluded patients with malignancy from our study. The inability to assess sarcopenia and malignancies in this study was one of the limitations. We also faced limitations due to the lack of data on the smoking status prevalence among COPD and non-COPD patients in our cohort.

## Conclusions

According to our findings, patients with COPD who also suffer from heart failure, stroke, chronic kidney disease, and dementia face a risk of prolonged ventilation that is more than five times higher compared to COPD without these comorbidities. It underscores the importance of implementing aggressive treatment and raising awareness about the heightened risk of prolonged ventilation among COPD patients with these specific comorbidities.

## Data Availability

The data that support the findings of this study are available from Kaohsiung Medical University Hospital but restrictions apply to the availability of these data, which were used under license for the current study, and so are not publicly available. Data are however available from the authors upon reasonable request and with permission of Kaohsiung Medical University Hospital.

## Abbreviations

aHR: Adjusted hazard ratios
COPD: Chronic obstructive pulmonary disease
CI: Confidence interval
HR: Hazard ratio
ICD-9-CM: International Classification of Diseases, Ninth Revision, Clinical Modification
TFP: Total follow-up period
